# Immunoexpression of CXCL12 and CXCR4 in giant cell granulomas of the jaws and giant cell tumor of bone

**DOI:** 10.4317/medoral.28299

**Published:** 2026-06-28

**Authors:** Vanessa Alves de Medeiros, Christany Rodrigues Ferreira, John Lennon Silva-Cunha, Éricka Janine Dantas da Silveira, Manuel Antonio Gordón-Núñez, Pollianna Muniz Alves, Cassiano Francisco Weege Nonaka

**Affiliations:** 1 Department of Dentistry, State University of Paraíba, Campina Grande, PB, Brazil; 2 Department of Biological and Health Sciences, Federal University of Western Bahia, Barreiras, BA, Brazil; 3 Department of Dentistry, Federal University of Rio Grande do Norte, Natal, RN, Brazil

**Keywords:** Giant cells, giant cell granuloma, giant cell tumor of bone, CXCL12, CXCR4.

## Abstract

**Background:**

Chemokines are proteins involved in various cellular processes; however, their participation in the pathogenesis of lesions containing multinucleated giant cells (MGC) has not been fully elucidated. This study investigated the immunoexpression of chemokine CXCL12 and its receptor CXCR4 in giant cell granulomas of the jaws (central giant cell granuloma [CGCG] and peripheral giant cell granuloma [PGCG]) and giant cell tumor of bone (GCTB).

**Material and Methods:**

Forty-five giant cell granulomas of the jaws (15 non-aggressive CGCG, 15 aggressive CGCG, and 15 PGCG) and 15 GCTB were selected. The percentages of cytoplasmic (CXCL12 and CXCR4) and nuclear (CXCR4) positivity in mononuclear cells (MC) and in non-cannibalistic (ncMGC) and cannibalistic MGC (cMGC) were determined.

**Results:**

All groups exhibited low median percentages of positivity for CXCL12 in MC (p>0.05). In ncMGC and cMGC, the highest median percentages of CXCL12 positivity were observed in GCTB (p>0.05). Cytoplasmic immunoexpression of CXCR4 was observed in all groups evaluated, with high median percentages of positivity in ncMGC and cMGC. Compared to non-aggressive CGCG, GCTB exhibited significantly higher cytoplasmic expression of CXCR4 in ncMGC (p<0.05). In MC, the highest median percentage of CXCR4 positivity was observed in GCTB, with a statistically significant difference compared to PGCG (p<0.05). All groups showed low median percentages of nuclear expression of CXCR4. Compared to PGCG, GCTB exhibited higher nuclear expression of CXCR4 in MC (p<0.05). Strong positive correlations were found between cytoplasmic and nuclear expression of CXCR4 in MC of non-aggressive CGCG, PGCG, and GCTB (p<0.05).

**Conclusions:**

The results suggest the potential involvement of CXCR4 in the pathogenesis of giant cell granulomas of the jaws and GCTB. This chemokine receptor may also contribute to differences in the biological behavior of these MGC-containing lesions. The relevance of CXCL12 for the development of the giant cell lesions studied appears to be variable.

## Introduction

Giant cell lesions are a group of diseases that share similar histopathological features but that differ in biological behavior and clinical manifestations. Central giant cell granuloma (CGCG) and peripheral giant cell granuloma (PGCG) are part of the spectrum of lesions that affect the jaws and are composed of mononuclear cells (MC) and osteoclast multinucleated giant cells (MGC) [1]. These cellular components are also present in giant cell tumor of bone (GCTB), a neoplasm that is more prevalent in the distal femur and proximal tibia [2,3].

Central giant cell granuloma is a benign intraosseous lesion of unknown etiology that has important clinical relevance because of its biological behavior, which ranges from relatively indolent to locally aggressive [4]. Mutually exclusive somatic mutations in TRPV4, KRAS, and FGFR1, which induce the activation of the mitogen-activated protein kinase (MAPK) signaling pathway, have been reported in up to 70% of cases of CGCG of the jaws [5]. On the other hand, GCTB is a locally aggressive neoplasm with potential for recurrence [3]. Its pathogenesis has been related to the p.Gly34Trp (G34W) mutation in the gene encoding histone H3.3 [6]. PGCG, in turn, is considered a reactive process located in the gingiva/alveolar ridge, which is caused by local irritation or tissue trauma [1,5].

Studies aimed at understanding the differences in the biological behavior of giant cell lesions, with a focus on CGCG and GCTB, have been conducted [3,7,8]. Within this context, studies investigating the participation of proteins involved in cell cycle [3], immune checkpoint [7], and extracellular matrix remodeling [8] have identified important differences between these lesions. Cellular cannibalism, a process whereby a cell engulfs another smaller cell in its cytoplasm, has been associated with the aggressiveness of CGCG and GCTB [9,10]. Despite advances in our understanding, the mechanisms underlying the differences in the biological behavior of giant cell lesions remain incompletely understood.

Chemokines are proteins that participate in various cellular processes, particularly cell migration [11]. When they bind to their receptors, chemokines can trigger cascades of events that produce diverse cellular responses. Chemokines are classified into four main subfamilies based on structural differences (C, CC, CXC, and CX3C) [11,12]. Among the members of the CXC subfamily, C-X-C chemokine ligand 12 (CXCL12) is particularly important because it is involved in a range of physiological and pathological processes [12]. This chemokine is able to bind to two types of receptors: C-X-C chemokine receptor 4 (CXCR4) and C-X-C chemokine receptor 7 (CXCR7) [11]. Although CXCL12 has a higher binding affinity for CXCR7, the latter is unable to stimulate G-proteins like CXCR4 does [13], requiring interaction with β-arrestin [11].

Studies have demonstrated the participation of chemokines and their receptors in various biological processes such as osteoclastogenesis, bone resorption [14], migration of osteoclast precursors [15], and tumorigenesis [11,13]. However, few studies have investigated the role of CXCL12 and CXCR4 in giant cell lesions, which were limited to GCTB [16,17]. Thus, there are important knowledge gaps regarding the participation of these proteins in the development of CGCG and PGCG (PubMed, accessed 24/04/2026).

Therefore, the present study aimed to evaluate the immunoexpression of CXCL12 and CXCR4 in giant cell granulomas of the jaws (CGCG and PGCG) and GCTB in an attempt to contribute to a better understanding of the pathogenesis and differences in the biological behavior of these lesions.

## Materials and Methods

*Sample*

The sample consisted of 45 cases of giant cell granulomas of the jaws (15 non-aggressive CGCG, 15 aggressive CGCG, and 15 PGCG) and 15 cases of GCTB. The CGCG and PGCG cases were obtained from the archives of the Oral Histopathology Laboratories of the Departments of Dentistry of the State University of Paraíba (UEPB) and the Federal University of Rio Grande do Norte (UFRN). The GCTB specimens were selected among cases stored at the Getúlio Sales Diagnósticos Laboratory (Natal, RN, Brazil). Only cases of CGCG, PGCG, and GCTB that had sufficient biological material for immunohistochemical analysis were included in the sample. For CGCG, only cases with clinical and radiographic information that permitted their classification as aggressive or non-aggressive were included [18]. Lesions previously submitted to conservative treatment were excluded from all groups. Recurrent lesions and specimens from patients with hyperparathyroidism or associated with syndromes based on family history and clinical, radiographic and laboratory examinations were also excluded. The clinical characteristics of PGCG and CGCG cases are shown in Table 1. The Institutional Ethics Committee on Research Involving Human Subjects approved the study (Protocol 70510823.1.0000.5187).

*Immunohistochemistry*

The selected samples, fixed in 10% formalin and embedded in paraffin, were cut into 3-μm-thick sections, which were mounted on silanized glass slides. The tissue sections were deparaffinized, rehydrated, and submitted to antigen retrieval with citrate buffer, pH 6.0, at 90ºC in a steamer for 60 min. For the blockade of endogenous tissue peroxidase, the sections were immersed in 3% hydrogen peroxide. After incubation with the primary antibodies against CXCL12 (dilution 1:50, clone P-159X; Santa Cruz Biotechnology, Dallas, TX, USA) and CXCR4 (dilution 1:200, clone 12G5; Santa Cruz Biotechnology, Dallas, TX, USA), the sections were washed in Tris-HCl buffer and treated with a dextran polymer-based complex (EnVisionTM Flex+, Dako North America Inc., Carpinteria, CA, USA). Peroxidase activity was visualized by immersing the sections in diaminobenzidine (EnVisionTM Flex DAB+, Dako North America Inc., Carpinteria, CA, USA). Finally, the tissue sections were counterstained with Harris hematoxylin, dehydrated, and coverslipped. Histological sections of human tonsils served as positive controls. For the negative control, the primary antibodies were omitted in the immunohistochemical protocol.

*Immunohistochemical analysis*

Immunoexpression of CXCL12 and CXCR4 was assessed quantitatively in MC and in cannibalistic (cMGC) and non-cannibalistic MGC (ncMGC) using an adaptation of the method described by Martini *et al*. [19]. Cells with smaller cell nuclei in their cytoplasm, totally or partially surrounded by a clear halo, were classified as cMGC [9]. For CXCL12, cytoplasmic immunoreactivity was assessed, while for CXCR4, cytoplasmic and nuclear immunoreactivity was analyzed separately. For both antibodies, brownish staining was defined as positive, regardless of intensity.

The slides were scanned and transformed into high-resolution digital images (MoticEasyScan Pro 6, Motic Inc., Richmond, BC, CAN). The images were viewed in the DSAssistant program (Motic Inc., Richmond, BC, CAN). A previously trained examiner, who was unaware of the clinicopathological data of the cases, performed the immunohistochemical analyses. First, areas of highest immunoreactivity to the antibodies were selected at 100× magnification (DSAssistant, Motic Inc., Richmond, BC, CAN). Next, five fields in these areas of highest immunoreactivity were photographed at 400× magnification (DSAssistant, Motic Inc., Richmond, BC, CAN). The ImageJ® program (Image Processing and Analysis in Java, National Institutes of Health, Bethesda, MD, USA) was used for the counting of immunostained and negative MC, cMGC, and ncMGC in each microscopic field. The values obtained for each field were summed and the percentages of positive MC, cMGC, and ncMGC were calculated.

*Statistical analysis*

The data were analyzed using the Jamovi Project program (version 2.6.24; Sydney, AU). The immunohistochemical results were submitted to the Shapiro-Wilk test, which revealed the absence of a normal distribution. Thus, the median percentages of CXCL12- and CXCR4-immunopositive cells were compared using the nonparametric Kruskal-Wallis test, followed by the Dwass-Steel-Critchlow-Fligner multiple comparisons post-test.

Differences in the immunoexpression of CXCL12 and CXCR4 according to cell type were evaluated using the nonparametric Wilcoxon signed-rank test. Spearman’s correlation test was applied to analyze possible correlations between the immunoexpression of these proteins. The magnitude of the correlation was interpreted as weak (<0.30), moderate (0.30-0.50), or strong (>0.50) according to Cohen’s classification [20]. For all statistical tests, a level of significance of 5% (*p<*0.05) was considered.

## Results

*Immunoexpression of CXCL12*

Cytoplasmic expression of CXCL12 in MC was more frequently observed in GCTB (60.0%) and aggressive CGCG (60.0%) (Table 2; Figure 1A-D). In ncMGC and cMGC, CXCL12 immunoreactivity was identified more frequently in GCTB and in a smaller proportion of non-aggressive CGCG (Table 2). All groups exhibited low median percentages of CXCL12 positivity in MC (*p>*0.05). In ncMGC and cMGC, the highest median percentages of positivity were identified in GCTB, with no statistically significant differences between groups (*p>*0.05) (Figure 2A).

*Immunoexpression of CXCR4*

Cytoplasmic expression of CXCR4 was observed in all CGCG, PGCG, and GCTB cases, both in MC and MGC (Table 2; Figure 1E-H), with high median percentages of positivity in ncMGC and cMGC (Figure 2B). In MC, the highest median percentage of immunopositivity for this protein was identified in GCTB, with a statistically significant difference when compared to PGCG (*p=*0.003) (Figure 2B). In ncMGC, GCTB exhibited significantly higher percentages of positivity than non-aggressive CGCG (*p=*0.037) (Figure 2B). No significant differences between groups were observed regarding the cytoplasmic expression of CXCR4 in cMGC (*p>*0.05) (Figure 2B).

Nuclear immunoexpression of CXCR4 was observed in MC of most GCTB (93.3%), aggressive CGCG (80.0%), non-aggressive CGCG (73.3%), and PGCG (60.0%) (Table 2; Figure 1E-H), with low median percentages of positivity. GCTB exhibited significantly higher percentages of nuclear expression of CXCR4 in MC when compared to PGCG (*p=*0.011) (Figure 2C).

In ncMGC, there was a higher frequency of nuclear expression in aggressive CGCG (93.3%) and non-aggressive CGCG (73.3%) (Table 2). In cMGC, a higher frequency of cases with nuclear immunoexpression of CXCR4 was observed in GCTB (86.7%), followed by aggressive CGCG (73.3%) (Table 2). There were no statistically significant differences in the nuclear expression of CXCR4 in ncMGC or cMGC between groups (*p>*0.05) (Figure 2C).

*Comparison of CXCL12 and CXCR4 immunoexpression according to cell type*

In all cell types of PGCG, non-aggressive CGCG, aggressive CGCG, and GCTB, the Wilcoxon signed-rank test showed significantly higher percentages of cytoplasmic expression of CXCR4 than CXCL12 (*p<*0.05). In all groups, there was no statistically significant difference in the immunoexpression of CXCL12 or CXCR4 between MGC types (*p>*0.05).

*Correlation between CXCL12 and CXCR4 immunoexpression*

The correlations between the immunoexpression of CXCL12 and CXCR4 according to cell type (MC, ncMGC, and cMGC) are summarized in Tables 3 and 4. Strong positive correlations were identified between cytoplasmic and nuclear immunoexpression of CXCR4 in MC of non-aggressive CGCG (*r=*0.814; *p<*0.001), PGCG (*r=*0.867 *p<*0.001), and GCTB (*r=*0.921; *p<*0.001). In PGCG, there was a strong positive correlation between the cytoplasmic and nuclear expression of CXCR4 in cMGC (*r=*0.733; *p=*0.002).

## Discussion

Studies have demonstrated the participation of the CXCL12/CXCR4 signaling pathway in the migration of osteoclast precursors, osteoclastogenesis, and bone resorption [15,21]; however, research on the involvement of this pathway in the pathogenesis of giant cell lesions is still scarce and limited to GCTB [16,17]. Within this context, the results of the present study corroborate the participation of CXCL12 and CXCR4 in the pathogenesis of GCTB and also suggest the potential involvement of these proteins, particularly of the chemokine receptor, in the development of CGCG and PGCG of the jaws.

Studies investigating GCTB reported expression of CXCL12 in both tissue specimens and cell cultures [16,17]. Liao *et al*. [17] identified CXCL12 concentrations compatible with physiological chemotactic levels in GCTB-conditioned media. The authors also observed a 2.5-fold higher migration rate of osteoclast precursors in response to GCTB-conditioned medium, suggesting that CXCL12 could be an important factor involved in the migration of osteoclast precursors in GCTB. Using gene expression analysis, De Vita *et al*. [16] found CXCL12 to be downregulated in three cases of GCTB. In the present study, although low median percentages of CXCL12 positivity were observed in GCTB, some cases exhibited considerable expression of this chemokine, especially in ncMGC and cMGC. Taken together, these findings suggest the involvement of CXCL12 in the pathogenesis of GCTB; however, its relevance in this process appears to be variable.

Similar to the results obtained for GCTB, the present study identified the expression of CXCL12 in CGCG and PGCG of the jaws, especially in MGC. These findings show for the first time the potential involvement of this chemokine in the pathogenesis of these lesions. In GCTB, a previous study demonstrated the role of stromal cell-derived CXCL12 in the migration of osteoclast precursors [17]. A similar function might be attributed to this chemokine in CGCG and PGCG of the jaws. Particularly the higher expression of CXCL12 in ncMGC and cMGC of GCTB, observed in the present study, is consistent with the more aggressive biological behavior of these lesions.

Previous studies on GCTB have identified significantly upregulated gene expression of CXCR4 [16], as well as immunoexpression of this protein in macrophages and osteoclasts [17]. In agreement with these results, the present study found high median percentages of cytoplasmic expression of CXCR4 in MC and MGC of GCTB. In particular, the higher immunoexpression of this chemokine receptor in MC of GCTB must be highlighted, a finding that might be related to the more aggressive behavior of this neoplasm when compared to CGCG and PGCG. On the other hand, the high median percentages of CXCR4 positivity in ncMGC and cMGC of CGCG and PGCG of the jaws also suggest an important role of this receptor in the pathogenesis of these lesions, possibly contributing to the migration of osteoclast precursors, osteoclastogenesis, and bone resorption [15,21]. However, differences in the biological behavior of aggressive and non-aggressive CGCG are probably not related to the expression of CXCR4.

The considerably higher levels of cytoplasmic expression of CXCR4 compared to CXCL12 identified in the present study indicate possible cytoplasmic functions of this receptor in CGCG, PGCG, and GCTB that are independent of ligand or surface binding. Within this context, Nengroo *et al*. [22] showed that overexpressed intracellular CXCR4 negatively regulates the expression of the proapoptotic protein death receptor 5 (DR5) in different human carcinoma cell lines and xenograft models. The authors found that this activity is independent of the presence of CXCL12 [22]. Furthermore, investigating the dynamics of CXCR4 activation and internalization, Perpiñá-Viciano *et al*. [23] suggested ligand-independent constitutive activity of this receptor. Taken together, these findings indicate functions of this receptor that are independent of the CXCL12/CXCR4 pathway or its surface expression [22].

Cellular cannibalism, a process characterized by the ability of a cell to engulf another living cell, has been linked to the aggressiveness of giant cell lesions [9,10]. Unlike phagocytosis, which is responsible for removing pathogens and cellular particles, the primary goal of cannibalism is to supply nutrients to the cell [9]. Within this context, cellular cannibalism may indicate high metabolic activity of MGC, especially in CGCG of the jaws and GCTB [9,10]. Furthermore, Barros *et al*. [9] suggested a greater osteolytic potential of cMGC in CGCG of the jaws.

Considering the previously described scenario, higher immunoexpression of CXCL12 and CXCR4 would be expected in cMGC of CGCG, PGCG, and GCTB. However, the present study revealed a similar expression profile of CXCL12 and CXCR4 in ncMGC and cMGC, suggesting that these proteins do not play fundamental roles in cellular cannibalism in CGCG, PGCG, or GCTB. It is therefore possible that CXCL12 and CXCR4 are primarily involved in the migration of osteoclast precursors, osteoclastogenesis, and bone resorption in these giant cell lesions. Consistent with this suggestion, the higher expression of CXCR4 in MC and of CXCL12 in ncMGC and cMGC of GCTB are compatible with the greater osteoclastic and aggressive potential of these lesions. Taken together, these findings highlight the need for further research to evaluate possible phenotypic and metabolic differences between cMGC and ncMGC in giant cell lesions.

Although incompletely understood, nuclear translocation of CXCR4 is an event that occurs in physiological and pathological processes, especially in malignant neoplasms [24-26]. This receptor directly interacts with proteins such as cyclophilin A and extracellular signal-regulated kinases (ERK), promoting the phosphorylation and nuclear translocation of extracellular signal-regulated kinase 1/2 (ERK1/2) [24]. Once in the nucleus, the latter modulates the expression of genes associated with cell survival [24]. Thus, despite the low median percentages of nuclear expression of CXCR4 observed in the present study, a possible contribution of the nuclear translocation of this chemokine receptor to the pathogenesis of CGCG, PGCG, and GCTB cannot be ruled out.

In the present study, strong and significant positive correlations between cytoplasmic and nuclear immunoexpression of CXCR4 were observed in MC of non-aggressive CGCG, PGCG, and GCTB. Similar results were found for cMGC of PGCG. These findings suggest that cytoplasmic accumulation of CXCR4 activates signaling pathways that culminate in the translocation of this receptor to the nucleus of MC and MGC. In MC, this process is particularly relevant considering the role of this cellular component in the pathogenesis of these lesions. Immunohistochemical and in situ hybridization studies of GCTB and CGCG have shown that MC are responsible for the formation of MGC through the recruitment, fusion, and differentiation of osteoclast precursors mediated by the expression of receptor activator of nuclear factor kappa B ligand (RANKL) [27,28].

Despite the important results found in this study, some limitations must be considered. The relatively small sample size may represent a limiting factor, as does the lack of follow-up and outcome data of the cases, including data on lesion recurrence and patient survival. Regarding GCTB, the lack of information on clinical stage is also an important limitation. Thus, further research is needed to broaden our understanding of the roles of CXCL12 and CXCR4 in the pathogenesis of giant cell lesions, as well as to identify any potential relationships between these proteins and the prognosis of CGCG and GCTB.

## Conclusions

In summary, our results suggest the potential involvement of CXCR4 in the pathogenesis of giant cell granulomas of the jaws and GCTB. This chemokine receptor may also contribute to the differences in the biological behavior of these MGC-containing lesions. On the other hand, the relevance of CXCL12 for the development of the giant cell lesions studied appears to be variable. 

## Figures and Tables

**Table 1:** Table 1: Clinical data of PGCG, non-aggressive CGCG, and aggressive CGCG.

**Clinical data**	**PGCG**	**Non-aggressive CGCG**	**Aggressive CGCG**
Age (in years)			
Range	7-71	6-78	10-59
Mean±standard deviation	35.3±17.2	35.1±20.0	25.6±18.0
Gender			
Female	6 (40.0%)	8 (53.3%)	5 (33.3%)
Male	9 (60.0%)	7 (46.7%)	10 (66.7%)
Anatomic location			
Maxilla	6 (40.0%)	6 (40.0%)	7 (46.7%)
Mandible	9 (60.0%)	9 (60.0%)	8 (53.3%)

**Table 2:** Table 2: Number of positive cases, median and range of the percentages of immunopositive MC, ncMGC and cMGC for CXCL12 (cytoplasm) and CXCR4 (nucleus and cytoplasm) in PGCG, non-aggressive CGCG, aggressive CGCG, and GCTB.

**Cell types/groups**	**CXCL12**		**CXCR4**			
			**Cytoplasm**		**Nucleus**	
	**Positive cases (%)**	**Median (range)**	**Positive cases (%)**	**Median (range)**	**Positive cases (%)**	**Median (range)**
MC						
PGCG	8 (53.3)	1.8 (0.0-51.8)	15 (100.0)	15.9 (0.7-86.8)	9 (60.0)	0.1 (0.0-31.4)
Non-aggressive CGCG	6 (40.0)	0.0 (0.0-11.0)	15 (100.0)	40.8 (2.0-91.0)	11 (73.3)	0.6 (0.0-69.1)
Aggressive CGCG	9 (60.0)	1.5 (0.0-30.8)	15 (100.0)	48.8 (1.8-98.6)	12 (80.0)	1.4 (0.0-31.4)
GCTB	9 (60.0)	4.6 (0.0-28.0)	15 (100.0)	86.8 (22.1-100.0)	14 (93.3)	3.1 (0.0-62.7)
ncMGC						
PGCG	7 (46.7)	0.0 (0.0-93.8)	15 (100.0)	95.1 (17.4-100.0)	9 (60.0)	1.1 (0.0-45.2)
Non-aggressive CGCG	4 (26.7)	0.0 (0.0-82.6)	15 (100.0)	93.8 (45.5-100.0)	11 (73.3)	4.6 (0.0-62.8)
Aggressive CGCG	6 (40.0)	0.0 (0.0-100.0)	15 (100.0)	100.0 (58.8-100.0)	14 (93.3)	3.1 (0.0-100.0)
GCTB	9 (60.0)	23.5 (0.0-100.0)	15 (100.0)	100.0 (80.0-100.0)	10 (66.7)	10.7 (0.0-78.0)
cMGC						
PGCG	7 (46.7)	0.0 (0.0-100.0)	15 (100.0)	94.6 (13.3-100.0)	9 (60.0)	0.7 (0.0-42.1)
Non-aggressive CGCG	4 (26.7)	0.0 (0.0-100.0)	15 (100.0)	100.0 (7.7-100.0)	11 (73.3)	1.2 (0.0-78.3)
Aggressive CGCG	4 (26.7)	0.0 (0.0-75.0)	15 (100.0)	100.0 (60.0-100.0)	11 (73.3)	2.3 (0.0-32.5)
GCTB	8 (53.3)	33.3 (0.0-100.0)	15 (100.0)	100.0 (80.0-100.0)	13 (86.7)	11.3 (0.0-68.8)

**Table 3:** Table 3: Spearman correlation coefficient (r) and statistical significance (p) of the immunoexpressions of CXCL12 and CXCR4 in PGCG, non-aggressive CGCG, aggressive CGCG, and GCTB, according to cell types.

**Cell types/proteins**	**PGCG**		**Non-aggressive CGCG**		**Aggressive CGCG**		**GCTB**	
	**r**	*p*	**r**	*p*	**r**	*p*	**r**	*p*
MC								
CXCL12 x CXCR4 (cytoplasm)	0.139	0.621	0.012	0.966	0.044	0.876	-0.059	0.835
CXCL12 x CXCR4 (nucleus)	0.220	0.431	-0.315	0.253	0.283	0.306	-0.059	0.835
CXCR4 (cytoplasm) x CXCR4 (nucleus)	0.867	<0.001	0.814	<0.001	0.362	0.185	0.921	<0.001
ncMGC								
CXCL12 x CXCR4 (cytoplasm)	-0.024	0.931	-0.025	0.928	-0.232	0.406	0.105	0.711
CXCL12 x CXCR4 (nucleus)	0.001	0.997	-0.234	0.402	-0.433	0.098	0.234	0.401
CXCR4 (cytoplasm) x CXCR4 (nucleus)	0.457	0.087	-0.155	0.582	0.060	0.831	-0.126	0.654
cMGC								
CXCL12 x CXCR4 (cytoplasm)	-0.085	0.763	-0.227	0.416	-0.024	0.934	-0.204	0.466
CXCL12 x CXCR4 (nucleus)	0.144	0.609	-0.193	0.490	-0.271	0.329	0.223	0.424
CXCR4 (cytoplasm) x CXCR4 (nucleus)	0.733	0.002	-0.017	0.952	-0.102	0.718	0.048	0.865

**Table 4:** Table 4: Spearman correlation coefficient (r) and statistical significance (p) of the immunoexpressions of CXCL12 and CXCR4 in PGCG, non-aggressive CGCG, aggressive CGCG, and GCTB, according to cell types.

**Cell types/proteins**	**PGCG**		**Non-aggressive CGCG**		**Aggressive CGCG**		**GCTB**	
	**r**	*p*	**r**	*p*	**r**	*p*	**r**	*p*
MC x ncMGC								
MC (CXCL12) x ncMGC (CXCR4 cytoplasm)	-0.075	0.789	-0.220	0.431	-0.212	0.448	-0.038	0.893
MC (CXCL12) x ncMGC (CXCR4 nucleus)	-0.095	0.736	-0.242	0.385	-0.113	0.690	0.359	0.189
MC (CXCR4 cytoplasm) x ncMGC (CXCL12)	0.207	0.459	-0.110	0.696	-0.117	0.678	-0.176	0.530
MC (CXCR4 nucleus) x ncMGC (CXCL12)	0.267	0.336	-0.446	0.095	-0.295	0.285	-0.187	0.504
MC x cMGC								
MC (CXCL12) x cMGC (CXCR4 cytoplasm)	-0.027	0.923	-0.060	0.832	0.085	0.763	-0.227	0.415
MC (CXCL12) x cMGC (CXCR4 nucleus)	0.084	0.767	-0.195	0.486	0.320	0.245	0.258	0.354
MC (CXCR4 cytoplasm) x cMGC (CXCL12)	0.215	0.442	-0.209	0.455	-0.197	0.481	-0.042	0.883
MC (CXCR4 nucleus) x cMGC (CXCL12)	0.287	0.300	-0.391	0.149	-0.184	0.512	-0.102	0.717

**Figure 1:**
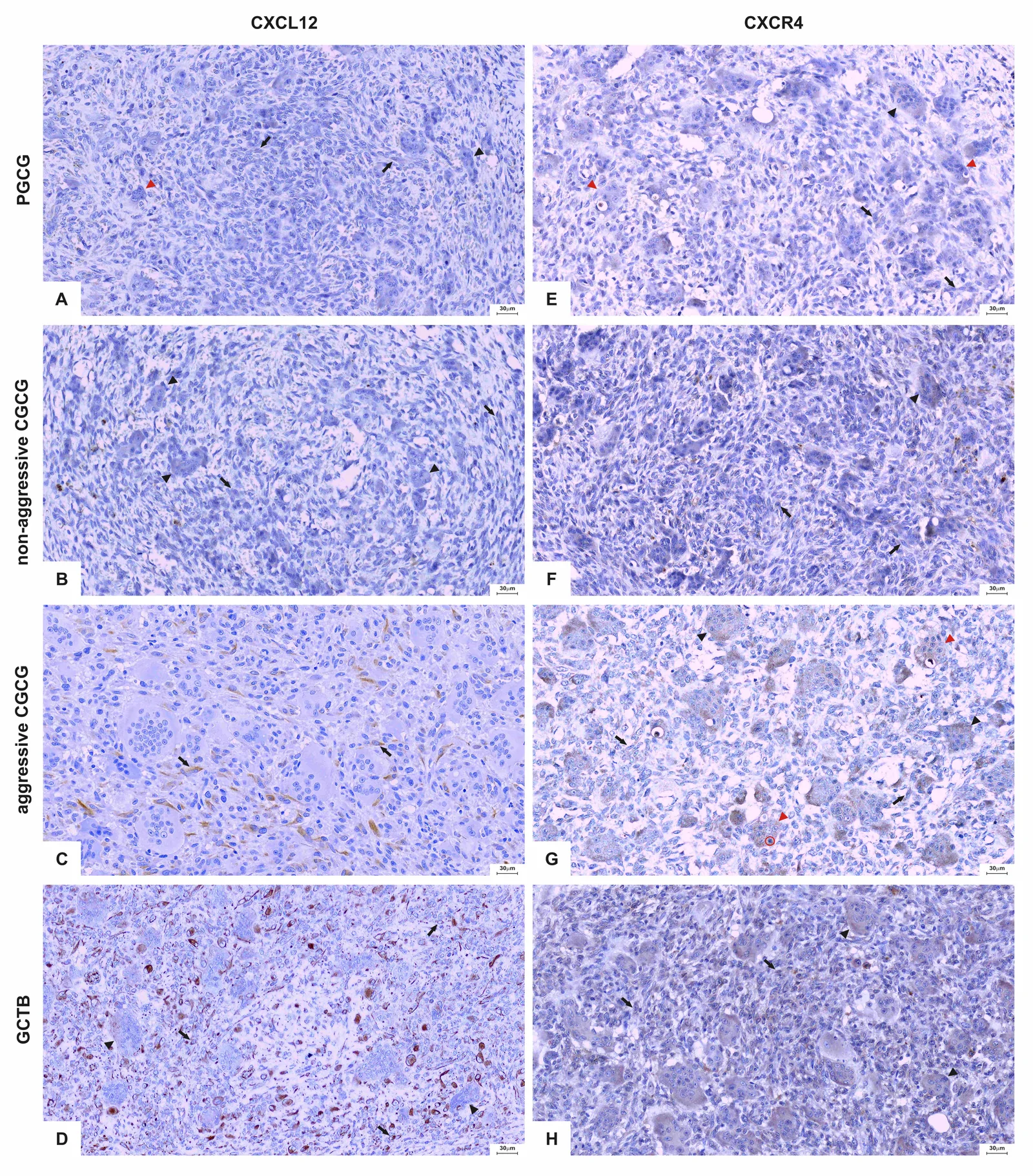
Fig. 1: Immunoexpression of CXCL12 and CXCR4 in PGCG, non-aggressive CGCG, aggressive CGCG, and GCTB. Cytoplasmic positivity for CXCL12 in MC (arrows), ncMGC (black arrowheads) and cMGC (red arrowheads) in PGCG (A), non-aggressive CGCG (B), aggressive CGCG (C), and GCTB (D) (EnVisionTM, scale bar: 30µm). Cytoplasmic immunoreactivity for CXCR4 in MC (arrows), ncMGC (black arrowheads) and cMGC (red arrowheads) in PGCG (E), non-aggressive CGCG (F), aggressive CGCG (G), and GCTB (H) (EnVisionTM, scale bar: 30µm). Nuclear positivity for CXCR4 in cMGC (red circle) of aggressive CGCG (G) (EnVisionTM, scale bar: 30µm).

**Figure 2:**
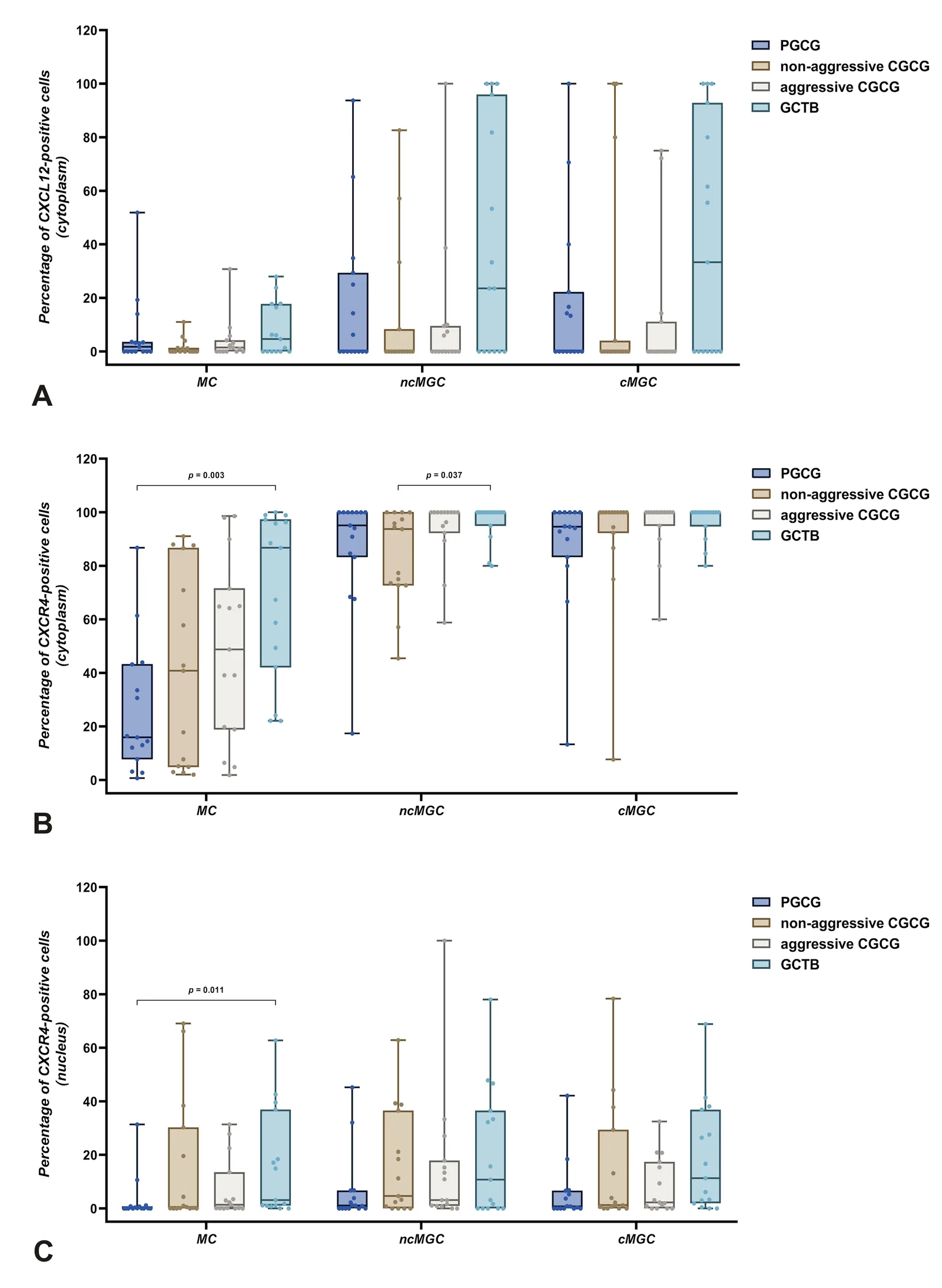
Fig. 2: Box and whisker plot charts illustrating the percentages of immunopositive MC, ncMGC and cMGC in PGCG, non-aggressive CGCG, aggressive CGCG, and GCTB. Cytoplasmic expression of CXCL12 (A). Cytoplasmic expression of CXCR4 (B). Nuclear expression of CXCR4 (C).

## Data Availability

None.
